# Primary hemiarthroplasty versus conservative treatment for comminuted fractures of the proximal humerus in the elderly (ProCon): A Multicenter Randomized Controlled trial

**DOI:** 10.1186/1471-2474-11-97

**Published:** 2010-05-25

**Authors:** Dennis Den Hartog, Esther MM Van Lieshout, Wim E Tuinebreijer, Suzanne Polinder, Ed F Van Beeck, Roelf S Breederveld, Maarten WGA Bronkhorst, Jan Peter Eerenberg, Steven Rhemrev, W Herbert Roerdink, Gerrit Schraa, Harm M Van der Vis, Thom PH Van Thiel, Peter Patka, Stefaan Nijs, Niels WL Schep

**Affiliations:** 1 Department of Surgery-Traumatology, Erasmus MC, University Medical Center Rotterdam, P.O. Box 2040, 3000 CA Rotterdam, the Netherlands; 2 Department of Public Health, Erasmus MC, University Medical Center Rotterdam, P.O. Box 2040, 3000 CA Rotterdam, the Netherlands; 3 Department of Surgery, Red Cross Hospital, P.O. Box 1074, 1940 EB Beverwijk, the Netherlands; 4 Department of Surgery, Bronovo Hospital, P.O. Box 96900, 2509 JH The Hague, the Netherlands; 5 Department of Surgery, Tergooi Hospitals, P.O. Box 10016, 1201 DA Hilversum, the Netherlands; 6 Department of Surgery, Medical Center Haaglanden, P.O. Box 432, 2501 CK The Hague, the Netherlands; 7 Department of Surgery, Deventer Hospital, P.O. Box 5001, 7400 GC Deventer, the Netherlands; 8 Department of Surgery, St. Jansdal Hospital, P.O. Box 138, 3840 AC Harderwijk, the Netherlands; 9 Department of Orthopedic Surgery, Tergooi Hospitals, P.O. Box 10016, 1201 DA Hilversum, the Netherlands; 10 Department of Surgery, Streekziekenhuis Koningin Beatrix, P.O. Box 9005, 7100 GG Winterswijk, the Netherlands; 11 Department of Traumatology, UZ Leuven, Herestraat 49, 3000 Leuven, Leuven, Belgium

## Abstract

**Background:**

Fractures of the proximal humerus are associated with a profound temporary and sometimes permanent, impairment of function and quality of life. The treatment of comminuted fractures of the proximal humerus like selected three-or four-part fractures and split fractures of the humeral head is a demanding and unresolved problem, especially in the elderly. Locking plates appear to offer improved fixation; however, screw cut-out rates ranges due to fracture collapse are high. As this may lead to higher rates of revision surgery, it may be preferable to treat comminuted fractures in the elderly primarily with a prosthesis or non-operatively. Results from case series and a small-sample randomized controlled trial (RCT) suggest improved function and less pain after primary hemiarthroplasty (HA); however these studies had some limitations and a RCT is needed. The primary aim of this study is to compare the Constant scores (reflecting functional outcome and pain) at one year after primary HA versus non-operative treatment in elderly patients who sustained a comminuted proximal humeral fracture. Secondary aims include effects on functional outcome, pain, complications, quality of life, and cost-effectiveness.

**Methods/Design:**

A prospective, multi-center RCT will be conducted in nine centers in the Netherlands and Belgium. Eighty patients over 65 years of age, who have sustained a three-or four part, or split head proximal humeral fracture will be randomized between primary hemiarthroplasty and conservative treatment. The primary outcome is the Constant score, which indicates pain and function. Secondary outcomes include the Disability of the Arm and Shoulder (DASH) score, Visual Analogue Scale (VAS) for pain, radiographic healing, health-related quality of life (Short-form-36, EuroQol-5D) and healthcare consumption. Cost-effectiveness ratios will be determined for both trial arms. Outcome will be monitored at regular intervals over the subsequent 24 months (1, 3 and 6 weeks, and 3, 6, 12, 18, and 24 months). Data will be analyzed on an intention to treat basis, using univariate and multivariable analyses.

**Discussion:**

This trial will provide level-1 evidence on the effectiveness of the two mostly applied treatment options for three-or four part and split head proximal humeral fractures in the elderly. These data may support the development of a clinical guideline for treatment of these traumatic injuries.

**Trial registration:**

Netherlands Trial Register (NTR2040).

## Background

Fractures of the proximal humerus are one of the most frequently encountered fractures in the elderly. The incidence is approximately 66 per 10,000 person years [1]. Most of these fractures are treated non-operatively and careful early motion with varying results. Especially, the treatment of three-or four-part fractures and split head humeral fractures is an unresolved problem [1-4].

Locking plates have been used during the last decade. They appear to offer improved fixation, however at considerable rates of complications such as non-union, malunion, or complaints of hardware (e.g., impingement). Also, the prevalence of screw cut-out ranges from 11 to 43% due to osteoporosis and avascular necrosis of the humeral head [5]. According to Hertel et al. [6] fractures of the proximal humerus involving the anatomical neck are mostly at risk for developing ischemia. Therefore, primary hemiartroplasty may be preferable over locking plates for these specific fracture types in the elderly.

Primary hemiarthroplasty and non-operative treatment of comminuted proximal humeral fractures have been described in a number of studies with varying functional results [7-15]. Up till now, only one RCT comparing HA with non-operative treatment has been published; less pain and better overall function was reported for the HA group (N = 32 patients) [13]. However, due to methodological limitations (e.g., indistinct inclusion criteria and differences in age at baseline) the outcome of this RCT may not be generalizable. A properly designed RCT is needed in order to gain insight into the best treatment for those comminuted proximal humeral fractures that are mostly at risk for avascular necrosis.

### Study objectives

The primary aim of this study is to compare the Constant score, reflecting functional outcome and pain, after primary hemiarthroplasty (HA) versus non-operative treatment in patients over 65 years of age who sustained a comminuted fracture of the proximal humerus.

Secondary aims are to determine the effect of primary HA versus non-operative treatment on the degree of disabilities of the arm, shoulder and hand (DASH score), level of pain (VAS), the rate of secondary interventions, complications and mortality, the radiographic healing, and the health-related quality of life (Short-Form 36, SF-36, and EuroQol-5D, EQ-5D) in these patients. Finally, upon calculation of the costs for both the HA and the non-operative groups, the cost-effectiveness of these treatments will be determined and compared.

Based upon limited literature data available, our main study hypothesis is that HA will result in higher Constant scores (reflecting better functional outcome with less pain) at 1 year compared with non-operative treatment of comminuted proximal humeral fractures in the elderly Despite higher initial costs, it is expected that HA will be more cost-effective than conservative treatment.

## Methods/Design

### Study design

The ProCon trial will follow a multicenter randomized controlled trial design. Eight centers in the Netherlands and one in Belgium will participate. The study started June 15, 2009. The trial has been designed in accordance with de Declaration of Helsinki (59th World Medical Association General Assembly, Seoul, October 2008) [16] and in accordance with the Medical Research Involving Human Subjects Act. It will follow the CONSORT (CONsolidation of Standards of Reporting Trials) guidelines [17,18].

### Recruitment and consent

Eligible persons presenting to the Emergency Department (ED) with a comminuted proximal humeral fracture will be informed about the trial at the ED. They will receive information and a consent form from the attending physician, the clinical investigator or a research assistant. After providing written informed consent, eligible patients will be randomized to two treatment strategies. Variable block randomization will be accomplished via a trial website. Allocation will be at random. Follow-up will take place over a period of two years.

### Study population

All persons aged 65+ who present at the Emergency Department with a comminuted fracture of the proximal humerus are eligible for inclusion. Presence of a proximal humeral fracture can be confirmed on X-ray, however fracture classification requires 3-dimensional Computed Tomography (CT) reconstructions [6,19].

**Figure 1:**
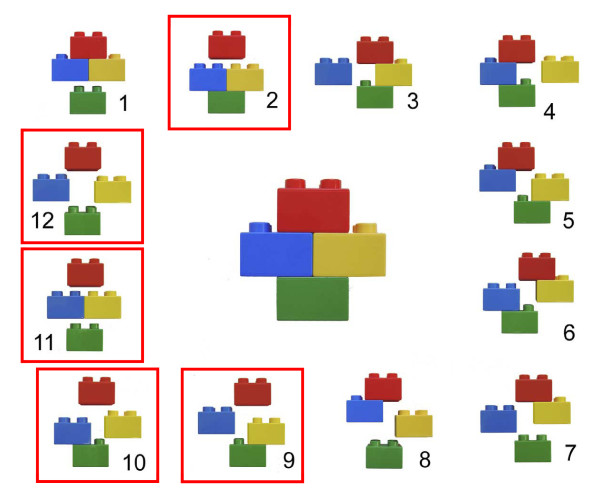
**Binary (LEGO) description system of the Hertel classification of proximal humerus fractures**. The 12 basic fracture patterns result after combining the 5 basic fracture planes. Basic fracture planes lie between the greater tuberosity and the head, the greater tuberosity and the shaft, the lesser tuberosity and the head, the lesser tuberosity and the shaft, and the lesser tuberosity and the greater tuberosity. There are 6 possible fractures dividing the humerus into two fragments, 5 possible fractures dividing the humerus into three fragments, and a single fracture dividing the humerus into four fragments Categories eligible for enrolment into the current trial are indicated in red boxes. **Reprinted from J Shoulder Elbow Surg, 13, Hertel R, Hempfing A, Stiehler M, Leunig M: Predictors of humeral head ischemia after intracapsular fracture of the proximal humerus, pp 427-433, with permission from Elsevier**.

**Figure 2:**
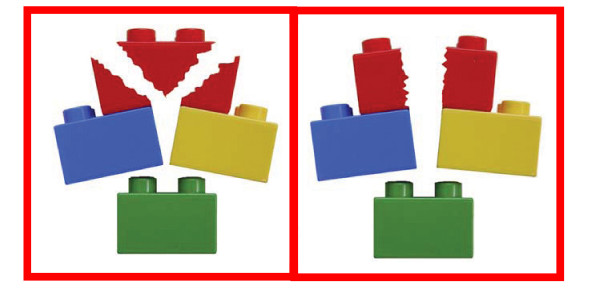
**Head-split components**. Classic head-split geometry (left) and special head-split geometry where both fragments remain perfused (right). Categories eligible for enrolment into the current trial are indicated in red boxes. **Reprinted from J Shoulder Elbow Surg, 13, Hertel R, Hempfing A, Stiehler M, Leunig M: Predictors of humeral head ischemia after intracapsular fracture of the proximal humerus, pp 427-433, with permission from Elsevier**.

Patients meeting the following inclusion criteria are eligible for enrolment:

1. Adult men or women aged 65 years and older (with no upper age limit)

2. Fracture of the humeral head

3. Selected three-part (Hertel classification type 9, 10, 11), selected four-part (Hertel type 12), anatomical neck (Hertel type 2), or split-head fractures of the humeral head in the judgement of the attending surgeon. All fractures should be classified according to the binary description system, based on 3D CT reconstructions (Figure 1 and 2)

4. Operative treatment within 21 days (if randomized for HA)

5. Provision of informed consent by patient

6. Assurance that the surgeon who will perform HA has attended the pre-trial HA course (Erasmus MC Skills lab)

If any of the following criteria applies, patients will be excluded:

1. Polytrauma patients

2. Patients with an additional traumatic injury of the affected arm

3. Patients with a pathological, recurrent or open humerus fracture

4. Patients with an impaired shoulder function (i.e., stiff or painful shoulder, neurologic disorder of the upper limb, or diagnosed rotator cuff impairment) prior to the injury

5. Retained hardware around the affected humerus

6. Patients with a disorder of bone metabolism other than osteoporosis (e.g., Paget's disease, renal osteodystrophy, osteomalacia)

7. Moderate or severe cognitively impaired patients (i.e., Mini-Mental Status Examination (MMSE) Six Item Screener with 3 or more errors)

8. Likely problems, in the judgment of the investigators, with maintaining follow-up (e.g., patients with no fixed address will be excluded)

9. Insufficient comprehension of the Dutch language to understand a rehabilitation program and other treatment information in the judgment of the attending physician

The Six Item Screener for dementia (exclusion criterion 7) is a brief and reliable instrument for identifying subjects with cognitive impairment. The patient is asked to remember three words (e.g., apple, table, penny), then to say the day of the week, month, year, and finally to recall the three words without prompts. Using a cut-off of 3 or more errors, the sensitivity and specificity of the Six Item Screener for diagnozing dementia was 88.7 and 88.0, respectively [20].

Exclusion of a patient because of enrolment in another ongoing drug or surgical intervention trial will be left to the discretion of the attending surgeon, on a case-by-case basis.

### Interventions

Patients will be randomized to either hemiarthroplasty or conservative treatment.

**Figure 3:**
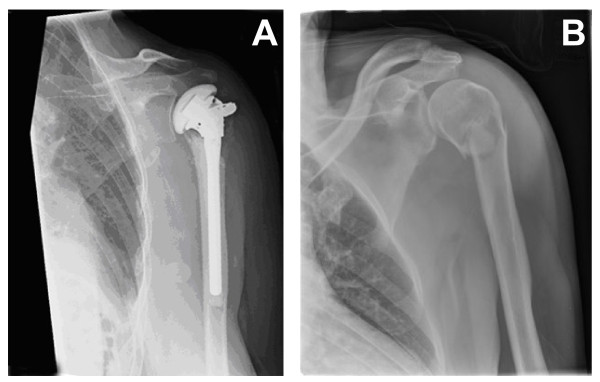
**Examples of proximal humerus fracture, managed by endoprosthesis (A) or conservative treatment (B)**.

Hemiarthroplasty (Figure 3A) will be performed using the Affinis^® ^Fracture shoulder endoprosthesis (Mathys AG Bettlach). No other prosthesis types will be used in order to increase homogeneity of data. Critical steps of the surgical procedure will be standardized. Standardized items will include the positioning of the patient (i.e., beach-chair position, with the scapula supported) and anesthesia (i.e., general or interscalene nerve block), surgical approach (a modified lateral deltoid split approach), exposure, shaft preparation, stem placement and cementing, and fixation of the tubercles with a cable wire [21]. After surgery, patients are allowed to use a sling for two days to one week. Patients will receive after-treatment following a standardized protocol, developed by an experienced physical therapist (Dept. of Rehabilitation and Physical Therapy, Erasmus MC, Rotterdam, The Netherlands). Anteflexion and elevation exercises may be started immediately if tolerated. Rotation exercises against resistance are not allowed during the first six weeks after surgery. Physical therapy sessions will be held at regular intervals, preferably two times a week during 12 weeks. The exact frequency and duration of physical therapy will largely depend upon the extent of functional recovery. This will be left at the discretion of the therapist.

In order to ensure similar peri-operative regimens, participating centers will standardize the following key aspects of pre-and post-operative care. Pre-operatively, patients will receive antibiotic prophylaxis (single dose) and thromboprophylaxis, and their condition prior to surgery will be optimized if necessary. Post-operatively, patients will receive thomboprophylaxis during hospital stay (e.g., unfractionated heparin, Low Molecular Weight Heparin (LMWH), or equivalent). Surgical delay and physical therapy and rehabilitation programs be recorded but not standardized.

Conservative treatment (Figure 3B) is defined by treatment with a collar and cuff for three weeks. After one week circumduction exercises will start under supervision of a physical therapist. At three weeks after trauma, full range of motion excercises will be initiated if tolerated. Physical therapy sessions will be held at regular intervals, preferably two times a week during 12 weeks. The exact frequency and duration of physical therapy will largely depend upon the extent of functional recovery. This will be left at the discretion of the therapist. Details of the therapy program will be recorded.

### Pre-trial hemiarthroplasty course

In order to ensure that all participating surgeons perform the surgical procedure in a similar manner, they will attend a one-day technique-oriented training prior to the commencement of the trial. The training will be held at the Surgical Skills Lab (Erasmus MC, Rotterdam, the Netherlands) and will be given by experienced surgeons (DDH, NWLS, SN). The surgical procedure will be practiced on human cadavers, with main focus on the critical aspects of the surgery including surgical approaches and implant-related insertion guidelines for the implant used. A refresher course will be planned after 6 months in order to ensure that sufficient knowledge is maintained. After each operation, surgeons will have to complete a surgical report form, in which space will be provided to report deviations, if any. Two experts (DDH and NWLS) will judge if surgery has been performed as intended and, if not, they will directly provide feedback to the surgeon.

### Outcome measures

The primary outcome measure will be the Constant score. This scoring system consists of four variables, reflecting both function and pain of the shoulder joint [22]. The subjective variables are pain (15 points), activities of daily living (ADL; i.e., sleep, work, recreation/sport; 10 points), and arm positioning (10 points), which give a total of 35 points. The objective variables are range of motion (ROM; 40 points) and strength (25 points), which give a total of 65 points. ROM includes forward flexion (10 points), lateral elevation (10 points), external rotation related to the head (10 points) and internal rotation related to the spine column (10 points). Forward flexion and lateral elevation are measured using a goniometer. Strength of abduction will be measured using a calibrated spring balance. The right and left shoulders will be assessed separately.

Secondary outcome measures are:

- Disabilities of the Arm, Shoulder and Hand (DASH) score

- Visual Analogue Scale (VAS) for pain

- Rates of secondary interventions

- Radiographic healing

- Mortality rate

- Complication rates

- Health-related quality of life: Short Form-36 (SF-36)

- Health Utility: EuroQol-5D (EQ-5D)

- Healthcare consumption (for cost calculations)

- Cost-effectiveness

The Disabilities of the Arm, Shoulder and Hand (DASH) Outcome Measure is a validated 30-item, self-report questionnaire designed to help describe the disability experienced by people with upper-limb disorders and also to monitor changes in symptoms and function over time [23-25]. The DASH outcome measure is scored in two components: the disability/symptom section (30 items, scored 1-5) and the optional high performance Sport/Music module (4 items, scored 1-5). The DASH disability/symptom score is a summation of the responses to 30 questions on a scale of 1 to 5. Scores may range from 0 points (no disability) to 100 points (severe disability). The questions test the degree of difficulty in performing a variety of physical activities because of arm, shoulder, or hand problems (21 items). They also investigate the severity of pain, activity-related pain, tingling, weakness, and stiffness (5 items), as well as the effect of the upper limb problem on social activities, work, sleep, and self-image (4 items).

Pain level will be determined using a 10-point Visual Analog Scale, in which 0 implies no pain and 10 implies the worst imaginable pain.

Secondary interventions within one year of initial treatment to promote fracture healing, relieve pain, treat infection, or improve function include the following:

1. (Reversed) Arthroplasty placement (conservative group only)

2. Plate fixation (conservative group only)

3. Incision and drainage for superficial surgical site infection (HA group only)

4. Incision and drainage for deep surgical site infection (HA group only)

5. Revision to Affinis^® ^Reversed prosthesis (HA group only)

6. Implant exchange (HA group only)

7. Implant removal (HA group only)

Radiographic healing will be determined using CT scanning at one year. Location and consolidation of the tubercles will be scored in duplicate by two experienced surgeons (DDH and NWLS). In case of differences between them, they will discuss the images until they reach an agreement.

Complications: complication rates in the HA group may include infection, neurovascular injury, malpositioning of the prosthesis, asceptic loosening of the prosthesis, dislocation of the tubercles. Complication rates in the control group may include malunion, nonunion, secondary dislocation, and symptomatic avascular necrosis of the humeral head.

The Short-Form 36 is a validated multi-purpose, short-form health survey with 36 questions, representing eight health domains that are combined into a physical and a mental component scale [26]. The Physical Component Scale (PCS) combines the health domains physical functioning (PF; 10 items), role limitations due to physical health (RP; 4 items), bodily pain (BP; 2 items), and general health perceptions (GH; 5 items). The Mental Component Scale (MCS) combines the health domains vitality, energy, or fatigue (VT; 4 items), social functioning (SF; 2 items), role limitations due to emotional problems (RE; 3 items), and general mental health (MH; 5 items). Scores ranging from 0 to 100 points are derived for each domain, with lower scores indicating poorer function. These scores will be converted to a norm-based score and compared with the norms for the general population of the United States (1998), in which each scale was scored to have the same average (50 points) and the same standard deviation (10 points).

The EuroQol (EQ-5D) is a validated and extensively used general health questionnaire to measure quality of life [27,28]. It is recommended for the assessment of QoL in trauma patients, especially for economic assessments [29,30]. EQ-5D has been developed by the EuroQoL Group in order to provide a simple, generic measure of health for clinical and economic appraisal [31]. EQ-5D consists of the EQ-5D descriptive system and the EQ Visual Analog Scale (EQ VAS). The EQ-5D descriptive system comprises five dimensions: mobility, self-care, usual activities, pain/discomfort, and anxiety/depression. Each dimension is marked as either no problems, some problems, or severe problems, which results in a 1-digit number expressing the level selected for that dimension. The digits for five dimensions are combined in a 5-digit number describing the respondent's health state. The EQ VAS records the respondent's self-rated health on a vertical, visual analog scale. Scores ranging from 0 ('Worst imaginable health state') to 10 ('Best imaginable health state') can be used as a quantitative measure of health outcome as judged by the individual respondents.

Cost measurement will be in accordance with Dutch guidelines for economic evaluations. Healthcare consumption data will be collected using a custom-made questionnaire. Health care costs will include costs of general practice care, medical specialist care, physical therapy, hospitalization, medication, home care, and other costs directly associated with diagnosis, treatment and rehabilitation. Where possible standard cost prices will be used as published by Oostenbrink will be used [32]. In accordance with guidelines for differential discounting, effects will be discounted at a rate of 1.5% and costs at 4% per year [33]. Results will be presented with 95% confidence intervals.

The incremental cost-effectiveness ratio of HA versus conservative treatment will be expressed in a cost-utility ratio, i.e., in terms of cost per Quality-Adjusted Life Years (QALY). Cost-utility ratios will be calculated by dividing the difference in mean costs of the two interventions by the difference in their mean effects. Policy makers and healthcare economists have proposed that costs varying from € 25,000 up to € 75,000 (US$ 31,600 - US$ 94,700) per QALY may be considered as acceptable [34,35].

In addition to the outcome variables mentioned above, the following data will be collected:

a) Intrinsic variables (baseline data): dominant side, age, gender, American Society of Anesthesiologists (ASA) grade, alcohol and tobacco consumption, comorbidity including osteoporosis, household composition, and medication use

b) Injury related variables: affected side, mechanism of injury, fracture classification (Hertel)

c) Intervention-related variables: surgical delay (i.e., time between fracture and surgery; HA-group only), time between injury and start of physical therapy, days of collar and cuff use, and total number of physical therapy sessions

### Follow up of patients

**Figure 4:**
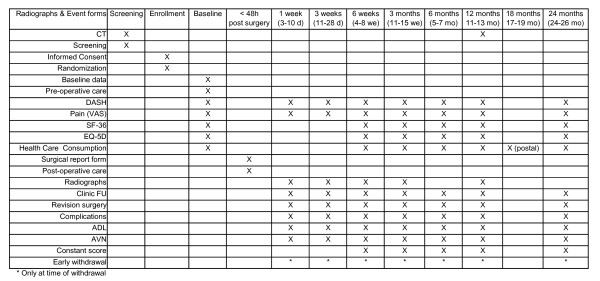
**Schedule of follow-up measurements**.

Patients will be followed for two years. In addition, at the 2-year follow-up (FU) visit, any secondary intervention planned will be recorded. A schedule of events is shown in Figure 4.

Clinical assessments will occur at the time of admission to the hospital (baseline), one week (3-10 days window), three weeks (11-28 days window), six weeks (4-8 weeks window), three months weeks (11-15 weeks window), six months (5-7 months window), 12 months (11-13 months window), and 24 months (24-26 months window) after surgery (HA group) or after start of conservative treatment (conservative group).

At baseline, multiple intrinsic (i.e., patient-related) and injury-related variables will be collected. At the three, six, 12, and 24 months FU visit, an independent researcher will determine the Constant score. In order to prevent bias the Constant score will be determined by an independent assessor. At each clinic FU visit, the researcher will ascertain patient status (i.e., secondary interventions, adverse events/complications, deaths), and will verify information within medical records. At each visit, patients will be asked to complete the questionnaires relating to disability (DASH score), and VAS for pain. From six weeks onwards, SF-36, EQ-5D, and health care consumption questionnaires will also be completed. A postal version of the latter questionnaire will be sent at 18 months in order to minimize recall bias.

As part of standard of care, X-rays of the shoulder will be made at the time of admission to hospital (baseline), at one, three and six weeks, and three and 12 months after surgery (HA group) or start of conservative treatment. CT scans that will be conducted pre-treatment, which are required for correct classification of the fracture, are also part of standard of care. As part of this trial, one additional CT-scan will be made at 12 months FU in order to determine consolidation (i.e. vanishing tubercles).

### Sample size calculation

Calculation of the required sample size is based on the assumption that the mean Constant score will be 47 in the conservatively treated patients and 56 in the HA group, assuming a SD of 12 for each group [10,14].

A two-sided test with an alpha level of 0.05 and a beta level of 0.2 requires 30 patients in every group. Anticipating a mortality rate of 10% in the first year due to a natural death rate in elderly patients [36], and a conservative dropout rate of 20% loss-to-follow up (based upon experience in previous trials), projects to a required sample size of 40 per group.

### Statistical analysis

The research data will be reported following the CONsolidated Standards of Reporting Trial (CONSORT). Data will be analyzed using the Statistical Package for the Social Sciences (SPSS) version 16.0 or higher (SPSS, Chicago, Ill., USA). Normality of continuous data will be tested with the Shapiro Wilk test, and homogeneity of variances will be tested using the Levene's test. Protocol violations will be recorded; data will be analyzed with and without patients with protocol violation.

Differences in baseline characteristics (i.e., intrinsic and injury-related variables) between both intervention groups will be assessed using the Student's T-test (parametric numeric data), Mann-Whitney U-test (non-parametric numeric data) or Chi-square test (categorical data). Data will be presented as mean ± SD (parametric data) or medians and percentiles (non-parametric data).

Univariate analysis will be performed to test the difference in primary and secondary outcome measures between the intervention groups using the Student's T-test (parametric data), Mann Whitney U-test (non-parametric data) or Chi-square analysis (categorical data).

Multinomial logistic regression analysis will be performed to model the relation between different covariates and the Constant score. Intrinsic and fracture-related variables that display a p-value < 0.5 in univariate analyses will be added as covariate. Similar models will be made to model the relation between covariates and the other outcome measures.

A p-value < 0.05 (2-sided tests) will be taken as threshold of statistical significance.

### Ethical considerations

The Medical Ethics Committee Erasmus MC acts as central ethics committee for this trial (reference number MEC-2009-178; NL26320.078.09). Approval has been obtained from the local Medical Ethics Committees in all participating centers. An information letter notifying the patients' participation will be sent to their general practitioners, unless a patient does not agree with this.

The Medical Ethics Committee Erasmus MC has given dispensation from the statutory obligation to provide insurance for subjects participating in medical research (Medical Research (Human Subjects) Compulsory Insurance Decree of 23 June 2003), because participation in this study is without risks.

## Discussion

The optimal treatment of three-or four part fractures and split head humeral fractures, with a high risk of developing head necrosis, is an unresolved problem in the elderly. The treatment of these fractures consists of conservative and operative strategies.

In general, hemiarthroplasty of the shoulder is indicated for the more complex fracture types; shoulder arthroplasty remains a valuable solution for the treatment of the non-reconstructable proximal humeral fractures in the elderly patients. A systematic literature review of 33 studies encompassing 1096 patients with three-or four-part proximal humeral fractures that used the Constant score as outcome measure, however, failed to proof superiority of HA over conservative treatment [37].

The results of this study will help clarify the question if primary HA is superior to non-operative treatment in selected proximal humeral fractures in the elderly. Higher Constant scores (reflecting better functional outcome with less pain) at one year are expected for the HA group. Better function and less pain may result in a better quality of life of patients. This may lead to a higher level of independency, and less health care consumption needs. Although costs for initial treatment will be higher in the HA-group (due to surgery) than in the non-operative group, it is expected that HA will be a cost-effective approach.

## List of abbreviations used

ADL: Activities of Daily Living; ASA: American Society of Anesthesiologists; CONSORT: CONsolidated Standards of Reporting Trial; DASH: Disabilities of the Arm, Shoulder and Hand (DASH) score; EQ-5D: EuroQol-5D; HA: Hemiarthroplasty; HR-QoL: Health-related quality of life; LMWH: Low Molecular Weight Heparin; MMSE: Mini-mental status exam; NTR: Netherlands Trial Registry (in Dutch: Nederlands Trial Register); ROM: Range Of Motion; QALY: Quality-Adjusted Life Years; QoL: Quality of Life; SF-36: Short Form 36; SPSS: Statistical Package for the Social Sciences; VAS: Visual Analog Scale.

## Competing interests

The authors declare that they have no competing interests.

## Authors' contributions

DDH, EMMVL, and NWLS developed the trial and drafter the manuscript. DDH will act as trial principal investigator. SN assisted in the design of the trial, including the chosen outcome measures. SP and EFVB assisted in the design of the healthcare consumption questionnaire and will perform the health economic analyses. WET, NWLS, and EMMVL will perform statistical analysis of the trial data. DDH, RSB, MWGAB, MMMB, JPE, SR, WHR, GS, EJTTH, HVDV, TPHVTL, PP, SN, and NWLS will perform the surgical procedures and will participate in patient inclusion and assessment. All authors have read and approved the final manuscript.

## Pre-publication history

The pre-publication history for this paper can be accessed here:

http://www.biomedcentral.com/1471-2474/11/97/prepub
